# The relationship of neurotrophin levels with stress-induced urinary incontinence in multiparous premenopausal women

**DOI:** 10.5339/qmj.2025.3

**Published:** 2025-02-13

**Authors:** Kübranur Ünal, Musa Latif Çöllüoğlu, Elif Erdem, Cansu Özbas¸, Özhan Özdemir

**Affiliations:** ^1^Department of Medical Biochemistry, Faculty of Medicine, Gazi University, Ankara, Turkey; ^2^Department of Medical Biochemistry, Lokman Hekim University, Ankara, Turkey; ^3^Department of Medical Biochemistry, Graduate School of Health Sciences, Gazi University, Ankara, Turkey; ^4^Department of Obstetrics and Gynaecology, Ankara Akyurt State Hospital, Ankara, Turkey; ^5^Department of Public Health, Faculty of Medicine, Gazi University, Ankara, Turkey; ^6^Department of Obstetrics and Gynaecology, Faculty of Medicine, University of Health Sciences, Ankara, Turkey *Correspondence: Kübranur Ünal. Email: kubranurunal@gazi.edu.tr

**Keywords:** Menopause, mixed urinary incontinence, multiparous, neurotrophins, stress urinary incontinence, urogynecology

## Abstract

**Objective:**

Urinary incontinence (UI) is involuntary urine leakage, mainly due to a feeling of high pressure in the abdominal part, the immediate and urgent need for micturition, or both. Neurotrophins (NTs) are a family of peptides that play a role in the regulation of nerve cells. Their effects on the lower urinary tract organs may provide a perspective to understand the development and diagnosis of UI. This study aims to investigate NT levels to understand how these molecules change in multiparous premenopausal women who suffer from stress-related UI. The study also evaluates diagnostic and distinguishing capabilities of NTs for these disorders.

**Methods:**

In this cross-sectional case–control study, multiparous premenopausal women underwent a urodynamic examination, a stress cough test, and were evaluated with an International Consultation on Incontinence Questionnaire-Short Form (ICIQ-SF). Participants were divided into three groups: 29 healthy women in the control group and two patient groups consisting of 26 women diagnosed with stress urinary incontinence (SUI) and 33 women diagnosed with mixed urinary incontinence (MUI). Nerve growth factor (NGF), brain-derived neurotrophic factor, neurotrophin-3 (NT-3), and neurotrophin-4 (NT-4) levels in serum were measured by enzyme-linked immunosorbent assay. The body mass index (BMI) and ICIQ-SF scores of the patients were also calculated. The data obtained were compared between the groups. Receiver-operating characteristic analysis was performed to determine the role of NTs in diagnosing UI.

**Results:**

The result showed that serum NGF and NT-3 levels were significantly low in both incontinence subtypes compared to the control group (*p* < 0.05). BMI scores and number of vaginal deliveries were higher in incontinence subtypes compared to the control group, and ICIQ-SF scores were higher in the MUI group.

**Conclusion:**

The differences in serum NGF and NT-3 levels were observed in multiparous premenopausal patients with UI. There was a decrease in serum NGF levels in MUI patients and serum NT-3 levels in SUI patients. Although the changes in serum NGF and NT-3 levels were significant, their discriminatory potential was weak or moderate.

## Introduction

Urinary incontinence (UI) is a condition that can cause urine leakage involuntarily.^1^ Stress urinary incontinence (SUI), urge urinary incontinence (UUI), and mixed urinary incontinence (MUI) are the most prevalent subgroups of UI. In SUI, complaints are related to urine loss due to high pressure in the abdominal part of the body when sneezing, coughing, straining, or pushing. In UUI, patients suffer from involuntary urine leakage with a feeling of immediate and urgent need for micturition. In MUI, a combination of SUI and UUI, patients complain of involuntary leakage of urine due to sneezing, coughing, straining, or pushing, accompanied by a sense of urgency.^2^

The lower urinary tract (LUT) includes functional units called the bladder and urethra, for storing and periodic elimination of urine.^3^ The proper functioning of the LUT organs is coordinated by the complex neuronal circuits of the nervous system. During bladder filling, norepinephrine provides an excitatory input to the smooth muscle of the urethra, but an inhibitory input to the smooth muscle in the body of the bladder through α1-adrenergic and β3-adrenergic receptors.^4,5^ When urinating, the activation of the parasympathetic nerve occurs through the muscarinic receptors (M2, M3) by acetylcholine (ACh), leading to bladder contraction, urethra relaxation, and urine outflow.^6,7^ Any damage or dysfunction of the elements of the nervous system of the LUT due to trauma, childbirth, aging, menopause, and high body mass index (BMI) can cause changes in the levels of molecules responsible for proper functioning and lead to improper urination and symptoms of UI.

Neurotrophins (NTs), one of the groups of molecules involved in the LUT function, are a family of peptides that bind exclusively to Trk receptors on the cell membrane of neural cells, causing receptor dimerization and autophosphorylation for activation. Following this activation, a downstream cascade for the survival, growth, differentiation, plasticity, and function of nerve cells is initiated.^8,9^ Nerve growth factor (NGF), brain-derived neurotrophic factor (BDNF), neurotrophin-3 (NT-3), and neurotrophin-4 (NT-4) are four of the well-known and most recognized members of the NT family. NGF is one of the well-studied NTs involved in the LUT function, especially bladder and related pathologies.^10,11,12^ BDNF is one of the other NTs that take part in voiding and neuronal regulation of bladder storage, in addition to its neurological effects on neuronal development, neuro-regeneration, depression, and pregnancy.^12,13^ NT-3 and NT-4, the other two NTs, are involved in changing the LUT function during neural injury and inflammation.^14^

Given their effects, NTs can be potential markers for understanding SUI and MUI. Therefore, we aimed to investigate the serum levels of NTs to determine how these potential biomarkers changed in multiparous premenopausal SUI and MUI patients compared to healthy controls. This study also examined the identification, diagnostic, and discrimination abilities of the levels of potential serum biomarkers for SUI and MUI. Furthermore, BMI scores, International Consultation on Incontinence Questionnaire-Short Form (ICIQ-SF) scores, and the number of births by both vaginal delivery (VD) and cesarean section (CS) were compared between groups to understand their effects on SUI and MUI.

## Materials And Methods

### Study design

This cross-sectional study was conducted in collaboration with the University of Health Sciences Gülhane Training and Research Hospital and Gazi University Hospital from August 2022 to April 2023. Participants were selected from the obstetrics and gynecology outpatient clinic of the former hospital, and samples were analyzed at the Department of Medical Biochemistry of the latter hospital.

### Ethical approval

Ethical approval for this study was granted by the Gazi University Clinical Research Ethics Board (dated July 25, 2022 and number 585). Participants were informed and signed written consent before their participation. All procedures and applications were performed in accordance with the Declaration of Helsinki.

### Participants

Participants included in this study were multiparous premenopausal women randomly selected from a urogynecology outpatient clinic with UI complaints. The participant's medical, surgical, gynecological, and obstetric history were examined. The demographic data of the participants were noted. The ICIQ-SF and the stress cough test (SCT) were administered to all participants by the urogynecologist. In the study by Hsu et al.,^15^ the SCT was found to be as an easy, quick, and inexpensive method with a sensitivity of 93.5% and a specificity of 90.0%. Participants with suspected SUI and MUI underwent urodynamic examination, considering their medical history, symptoms, and ICIQ-SF results. Based on these evaluations, the participants were divided into three groups. A control group consisted of 29 healthy women whose SCT was negative and ICIQ-SF scores were 0. Patients with UI were divided into two subgroups according to the urodynamic examination, SCT, and the answer to the sixth question of the ICIQ-SF: 26 women of SUI patients and 33 women of MUI patients. The sample size was determined using the power analysis program (G Power Statistical Programme version 3.1.9.4, Universität Düsseldorf, Germany). It was determined that 40 people would be sufficient to test the null hypothesis.

### Questionnaire

In the study by Çetinel et al.^16^ entitled “The validation study of ICIQ-SF Turkish version”, self-administrated ICIQ-SF for Turkish was validated. First, they carried out the translation of the questionnaire from English to Turkish by bilingual native speakers. Then, a pilot study was conducted to test internal consistency (reliability) and stability (test–retest reliability). They found that in the internal consistency assessment, the Cronbach alpha coefficient was 0.71, showing adequate internal consistency, and the test–retest assessment was 0.98 for the third question, 0.95 for the fourth question, and 0.97 for the fifth question (*p* < 0.001). In our study, the ICIQ-SF validated for Turkish was applied to the participants to evaluate the effects of UI on their lives. The first and second items were asked for demographic purposes. The third, fourth, and fifth items addressed the frequency of urine leakage, the intensity of urine leakage, and the quantification of urine leakage, respectively. The sixth item consisted of multiple choices to understand the type of UI, whether SUI or MUI. According to the answers to the third, fourth, and fifth items, the score of the patients ranged from 0 to 21 – the higher the score, the greater the impact of UI on daily life.

### Exclusion criteria

The criteria for patients excluded in this study are as follows: being menopausal, being grand multiparous, having mental retardation and psychological disorders, having systemic inflammation, having a BMI of 35 at any time in their life, without becoming pregnant, being diagnosed with bladder or invasive cervical cancer, excessive caffeine consumption, having Cushing's disease, neurological disorders, being diagnosed with fibroids or pelvic organ prolapse pressing on the bladder, having a history of open abdominal surgery for nothing other than the CS or non-CS section with midline incision, and having skin diseases. NT levels may be affected by neuropathy, diabetes mellitus, and antihypertensive drugs with diuretics. Therefore, patients were selected to exclude these potentially confounding diseases and drugs. Patients with SUI and MUI were included in the study, and patients with other types of incontinence were excluded from the study.

### Sample collection

The serum samples used in this study were selected from outpatients for routine control. Fasting blood samples were collected in the morning after ICIQ-SF, SCT, and urodynamic evaluation. With the consent of each participant, 10 ml of blood was taken from their antecubital vein into serum separator tubes. After clotting for 30 minutes at room temperature, the tubes were centrifuged at 1,300g for 10 minutes to obtain the separated serum. Eppendorf tubes were used to divide the separated serum samples. They were stored at -80°C until the start of the study. The frozen serum samples were thawed at 2–8°C before analysis. Serum samples with hemolysis and icterus were excluded.

### Measurement of serum levels of investigated biomarkers

The serum level measurements of NGF, BDNF, NT-3, and NT-4 were performed using enzyme-linked immunosorbent assay (ELISA) kits (ELK Biotechnology Co., Ltd, Wuhan, China). The working principle of the kit mentioned was based on a sandwich enzyme immunoassay. The color change was measured with a microplate reader (ELx800 UV Universal Microplate Reader, Bio-Tec. Instruments, Inc.) at a wavelength of 450 nm ± 10 nm. The concentrations of the target biomarkers were determined by comparing their optical density values with standard curves. The detection limits of the ELISA kit for serum NGF, BDNF, NT-3, and NT-4 were 15.63–1,000 pg/mL, 31.25–2,000 pg/mL, 78.13–5,000 pg/mL, and 15.63–1,000 pg/mL respectively. The intra-assay and inter-assay coefficients of variation were < 8% and < 10%, respectively, for NGF, BDNF, NT-3, and NT-4.

### Statistical analysis

The statistical package program SPSS 23.0 was used to evaluate the research data. Median (IQR (Interquartile Range) 25–75), frequency distribution, and percentage were used to present descriptive statistics. The Kruskal–Wallis test for multiple comparisons and the Mann–Whitney test for comparing two groups were used because parametric test conditions were not applicable for the continuous variables. Receiver-operating characteristic (ROC) analysis was performed to determine the role of BDNF, NT-3, NT-4, and NGF in the diagnosis of UI and the diagnosis of different types of UI (SUI and MUI) and to determine the cut-off point for these parameters. When evaluating the areas under the curve, the values 0.90–1.0 were considered “excellent”, 0.80–0.89 “good”, 0.70–0.79 “moderate”, 0.60–0.69 “weak”, and 0.50–0.59 “unsuccessful”. The cut-off point was determined for the parameters with moderate, good, and excellent discrimination. The Youden index was used to determine the cut-off point. The statistical significance value was accepted at *p* < 0.05 for all statistics.

## Results

### Serum NT levels

**Figure 1:**
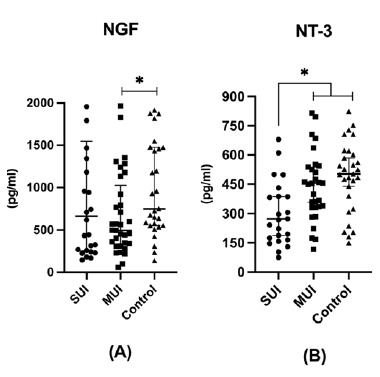
Serum NGF (A) and NT-3 (B) levels of the study groups. * indicates a significant difference between groups. SUI: stress urinary incontinence, MUI: mixed urinary incontinence, NGF: nerve growth factor, NT-3: neurotrophin-3.

Data on the serum NT values of the study groups are shown in Table 1. The serum NGF levels of the MUI group were found to be significantly low compared to the control (*p* < 0.05) (Figure 1A), and serum NT-3 levels were found to be significantly low in the SUI group compared to the MUI (*p* < 0.05) and control groups (*p* < 0.05) (Figure 1B). There was no significant difference between the SUI, MUI, and control groups in terms of serum BDNF and NT-4 levels. BMI and VD were identified as potential confounding variables that could influence serum NT levels. To avoid biasing the results, statistical analyses were conducted with adjustments for these factors, thereby ensuring that their effects were controlled for when evaluating the primary outcomes.

### Clinical characteristics

The clinical characteristics of the study groups are shown in Table 2. The BMI scores of SUI (*p* < 0.05) and MUI (*p* < 0.05) patients were significantly high compared to the control group. Moreover, the ICIQ-SF score of the MUI group was significantly high compared to the SUI group (*p* < 0.05). The number of VD was found to be significantly high in the SUI (*p* < 0.05) and MUI (*p* < 0.05) groups compared to the control group. There was no significant difference between SUI, MUI, and control groups with regard to the number of CS, the number of women giving birth by only CS, only vaginal birth, and both VD and CS (*p*>0.05).

### ROC curves

**Figure 2:**
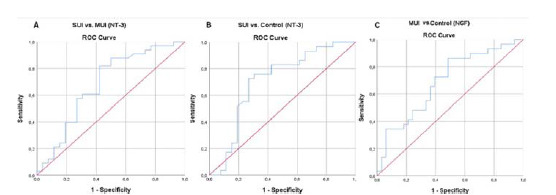
ROC curve of SUI versus MUI for NT-3 (A), SUI versus control for NT-3 (B), and MUI versus control for NGF (C). ROC: receiver-operating characteristic; SUI: stress urinary incontinence, MUI: mixed urinary incontinence, NGF: nerve growth factor, NT-3: neurotrophin-3.

The ROC analysis of serum biomarker levels for the SUI, MUI, and control groups is shown in Table 3. When comparing the ROC curves of BDNF, NGF, NT-3, and NT-4 for the SUI and MUI groups, the area under the curve (AUC) of NT-3 was found to be 0.680 (95% confidence interval (CI): 0.537–0.823, *p* = 0.018), which was significant and higher than the AUC of the other three NTs (Figure 2A). The cut-off point of NGF was 308 (sensitivity: 81.8%, specificity: 57.7%). When comparing the ROC curves of BDNF, NGF, NT-3, and NT-4 for the SUI and control groups, it was observed that the AUC of NT-3 was 0.704 (95% CI: 0.557–0.850, *p* = 0.010), which was significant and higher than the AUC of the other three NTs (Figure 2B). The cut-off point of NT-3 was 435 (sensitivity: 73.1%, specificity: 60.6%). When comparing the ROC curves of BDNF, NGF, NT-3, and NT-4 for the MUI and control groups, it was observed that the AUC of NGF was 0.690 (95% CI: 0.557–0.822, *p* = 0.010), which was significant and higher than the AUC of the other three NTs (Figure 2C). The cut-off point of NGF was 575 (sensitivity: 60.6%, specificity: 72.4%).

## Discussion

UI is a common disease among women, described as complaints of involuntary leakage of urine.^1^ SUI and MUI are the most prevalent subtypes of UI.^17^ In our study, we aimed to evaluate how the serum levels of NT biomarkers, BMI scores, ICIQ-SF scores, type of delivery, and their numbers change for the SUI, MUI, and control groups. Furthermore, ROC analysis was performed to evaluate the discriminatory ability of the investigated markers to distinguish SUI, MUI, and healthy individuals.

The literature review found that the NTs NT-3 and NT-4 were not as much focused as compared to NGF and BDNF in LUT dysfunction. To our knowledge, this is the first study to evaluate the serum levels of NT-3 and NT-4, as well as BDNF and NGF in SUI and MUI patients.

Although many studies have measured urinary NGF levels, measurements of serum NGF level are rare. Liu et al. measured urinary and serum NGF levels in patients with interstitial cystitis/bladder pain syndrome (IC/BPS).^18^ Their results showed that an increase in both urinary and serum NGF levels. However, the increase in serum NGF levels was not related to the causes of IC/BPS but rather to the result of medical comorbidities. On the contrary, another study by Liu et al. showed that urinary NGF levels can be used as a biomarker in MUI patients with DO (Detrusor Overactivity).^19^ In contrast to these studies, our study found a significant decrease in serum NGF levels in MUI patients compared to the control group. Considering the above-mentioned studies and our results together, we can speculate that the decrease in serum NGF levels, in addition to the increase in urinary NGF levels, may provide an idea for the diagnosis of MUI in multiparous premenopausal women.

Studies to understand the relationship between NTs and SUI have focused mainly on BDNF.^20–23^ To mimic SUI, a model organism was created by dual-simulated childbirth injury consisting of nerve and muscle injuries. A study by Balog et al. when investigating regenerative electrical stimulation found that although there was a difference in tissue expression of BDNF and NT-4 levels measured immunohistochemically, there was no significant difference in serum BDNF and NT-4 levels.^24^ Similarly, the present study did not find a significant difference in serum BDNF and NT-4 levels between the study groups. It may be concluded that even if BDNF is involved in the recovery of SUI, it may not be suitable as a biomarker for diagnosis.

Research studies investigating the effect of NT-3 on UI are rare. The study by Bagin' ska et al. examined urinary levels of NTs in children and investigated the effect of treatment with transcutaneous electrical nerve stimulation (TENS) for overactive bladder (OAB).^25^ Their results showed that although NT-3 levels were low in the intervention group and high in the control group before TENS treatment, they observed an inverse relationship after normalization with creatinine (Cr). Therefore, based on suggestive data, they speculated that low NT-3 levels may play a role in underactive bladder (UAB) syndrome and detrusor underactivity (DU). In another study by Dewulf et al., the presence of SUI in patients with DU may be considered a factor in the diagnosis of UAB.^26^ We also found that serum NT-3 levels were significantly low in both the MUI and control groups. Based on the two studies mentioned above, it can only be hypothesized that low NT-3 levels in SUI patients may be an indicator of UAB if accompanying DU is present.

Increased BMI scores can be considered a risk factor for UI and its subtype.^27^ A study by Townsend et al. demonstrated that increasing BMI can be considered an independent risk factor for the development of incontinence.^28^ Another study by Hunskaar suggested that weight gain in obesity could exert an effect similar to pregnancy.^29^ Similarly, the present study showed, in accordance with previously mentioned studies, a significant increase in BMI scores of the SUI and MUI groups compared to the control group.

The ICIQ-SF is a validated questionnaire for assessing UI in terms of frequency, volume, and impact on quality of life (QoL). Additionally, the increase in ICIQ-SF scores was positively correlated with the severity of UI.^30^ In a retrospective study by Dedicação et al., the impact of the type of UI on the QoL of women was compared. They found that the negative impact of MUI on QoL was more significant among incontinence patients.^31^ Similarly, we found that the ICIQ-SF scores of the MUI group were significantly higher than those of the SUI group. This result indicates that the severity and interference of UI in daily life may be higher in MUI patients than in SUI patients.

Women's obstetric history can have a significant impact on their continence mechanism.^32^ Pregnancy and childbirth can increase the risk of incontinence.^33^ However, it is speculated that the mode of delivery affects the risk of UI differently. The study by Hannah et al. showed that multiparous women were at higher risk of UI compared to primiparous women, and CS may reduce the risk of UI compared with VD regardless of parity.^34^ Another study by Barbosa et al. investigating the two-year postpartum prevalence of UI found that the delivery method had no association with pelvic floor muscle dysfunction and the prevalence of UI. Furthermore, the CS did not affect protection against UI two years after delivery.^35^ In our study, similar to that of Hannah et al., the number of VD was higher in the SUI and MUI groups than in the control group. According to our results, an increase in the number of VD may increase the risk of incontinence in both SUI and MUI. On the contrary, in accordance with Barbosa et al., no effect of the number of CS on SUI and MUI was observed.

The diagnosis of UI is based on symptomatic and urodynamic observations.^36^ However, the presence of various NTs in urine samples of some UI subtypes led to the emergence of opinions about the use of these molecules in diagnosis. In the study by Vijaya et al., ROC analyses were performed to determine the diagnostic value of NGF and predict incontinence types. They showed that the discriminatory ability of urinary NGF/Cr measurement was weak in different symptomatic groups except for OAB and non-OAB patients.^37^ In our study, ROC analyses were performed to determine the diagnostic value of biomarkers and the prediction of incontinence types. We found significant differences in NGF and NT-3 that could predict diagnostic incontinence types. In contrast to the previously mentioned study, serum NGF had a weak diagnostic value in detecting MUI compared to the control group. Likewise, serum NT-3 had a poor diagnostic value in detecting SUI and MUI. However, serum NT-3 provided a moderate diagnostic value in the diagnosis of SUI compared to the control group.

Our study has several strengths that increase the significance of the results and may serve as a guide for further studies in this field. First, in this study, all procedures and examinations were performed under the supervision of a urogynecologist. Second, the selection of participants was based not only on one criterion, but also on several criteria such as the ICIQ-SF, the SCT, and, if necessary, a urodynamic test to ensure that the individuals selected for the study belonged to the right group. Third, the study evaluated four of the most commonly studied NTs, providing a multifaceted understanding of these molecules in UI. Finally, a multidisciplinary approach combining obstetrics and gynecology with biochemistry enriches the scope and depth of the research.

Our study also faced several limitations. The first limitation was urinary NT levels. Measuring urinary levels of target biomarkers can provide a broad perspective to evaluate their diagnostic value. Another limitation is the age range of the participants. The narrow age range between 40 and 50 years limits the generalizability and utility of NTs as a biomarker. Increasing the age intervals could provide more solid evidence for the use of NTs as a biomarker. A further limitation of this study was the small number of recruited subjects. Increasing the number of participants could contribute to more comprehensive and credible results for statistical analysis.

## Conclusion

Our study demonstrated the changes in serum NT levels of multiparous premenopausal patients with UI. Although the decrease in serum NGF levels in MUI patients and serum NT-3 levels in SUI patients were significant, their discriminatory ability was weak or moderate. On the contrary, increased BMI scores and number of VD in SUI and MUI patients can be considered as confirmation of the effect of obesity and delivery on UI. In addition to the main methods for identifying SUI and MUI in patients, measurement of serum NTs can provide an alternative, straightforward, quick, and inexpensive way of diagnosis. However, future research is needed to understand the actual impacts of serum NTs on UI and their true potential in differentiating and detecting SUI and MUI.[Table tbl4]

## Figures and Tables

**Table 1:** Serum NT values of the study groups.

Clinical characteristics	SUI (*n* = 26)	MUI (*n* = 33)	Control (*n* = 29)	*p*

NGF (pg/ml)	664.3 (254–1,547)	496.9 (327–1,025)^‡^	751.8 (553–1,476)	0.010^‡^

BDNF (pg/ml)	42.1 (34–94)	44.1 (35–103)	39.4 (31–50)	>0.05

NT-3 (pg/ml)	284.6 (172–499)^†^	455.9 (334–594)^‡‡^	502.9 (357–611)	>0.010^†^ 0.018^‡‡^

NT-4 (pg/ml)	634.3 (278–1,077)	441.2 (178–1,087)	378.3 (197–679)	>0.05


SUI: stress urinary incontinence, MUI: mixed urinary incontinence, NGF: nerve growth factor, BDNF: brain-derived neurotrophic factor, NT-3: neurotrophin-3, NT-4: neurotrophin-4.

^†^The value is significantly different between SUI and the control group.

^‡^The value is significantly different between MUI and the control group.

^‡‡^The value is significantly different between the MUI and SUI groups.

**Table 2:** Clinical characteristics of the study groups.

Clinical characteristics	SUI (*n* = 26)	MUI (*n* = 33)	Control (*n* = 29)	*p*

BMI (kg/m^2^)	28.2 (27.2–29.4)^†^	28.3 (26.3–31.2)^‡^	26.3 (23.1–28.8)	>0.024^†^ 0.009^‡^

ICIQ-SF score	6.0 (4–9)	12.0 (9–15)^‡‡^	–	< 0.001^‡‡^

Type of delivery				

Number of VD	2 (2–3)^†^	2 (2–3)^‡^	2 (1.25–2)	>0.015^†^ 0.018^‡^

Number of CS	2 (2–3)	2 (1–3)	1.5 (1–2)	>0.05

Only CS (n, %) Yes No	5 (19%) 21 (81%)	8 (24%) 25 (76%)	5 (17%) 24 (83%)	>0.05

Only VD (n, %) Yes No	18 (69%) 8 (31%)	22 (67%) 11 (33%)	17 (58%) 12 (42%)	>0.05

Both VD and CS (n, %) Yes No	3 (12%) 23 (88%)	3 (9%) 30 (91%)	7 (24%) 22 (76%)	>0.05


SUI: stress urinary incontinence, MUI: mixed urinary incontinence, BMI: body mass index, ICIQ-SF: International Consultation on Incontinence Questionnaire-Short Form, VD: vaginal delivery, CS: cesarean section.

^†^The value is significantly different between SUI and the control group.

^‡^The value is significantly different between MUI and the control group.

^‡‡^The value is significantly different between the MUI and SUI groups.

**Table 3:** ROC curve analysis of serum NT biomarkers between the study groups.

	**SUI vs. MUI**	**SUI vs. control**	**MUI vs. control**

ROC analysis	**NT-3**	**NT-3**	**NGF**

AUC (95% CI)	0.680 (0.537–0.823)	0.704 (0.557–0.850)	0.690 (0.557–0.822)

*p*	0.018	0.010	0.010

Cut-off (pg/ml)	308	435	575

Sensitivity (%)	81.8	73.1	60.6

Specificity (%)	57.7	60.6	72.4


ROC: receiver-operating characteristic, SUI: stress urinary incontinence, MUI: mixed urinary incontinence, NT-3: neurotrophin-3, NGF: nerve growth factor, AUC: area under the curve, CI: confidence interval.

**Table tbl4:** List of abbreviations

ACh	Acetylcholine

AUC	Area Under the Curve

BDNF	Brain-Derived Neurotrophic Factor

BMI	Body Mass Index

CI	Confidence Interval

Cr	Creatinine

CS	Cesarean Section

DO	Detrusor Overactivity

DU	Detrusor Underactivity

ELISA	Enzyme-Linked Immunosorbent Assay

IC/BPS	Interstitial Cystitis/Bladder Pain Syndrome

ICIQ-SF	International Consultation on Incontinence Questionnaire-Short Form

LUT	Lower Urinary Tract

MUI	Mixed Urinary Incontinence

NGF	Nerve Growth Factor

NT	Neurotrophin

NT-3	Neurotrophin-3

NT-4	Neurotrophin-4

OAB	Overactive Bladder

QoL	Quality of Life

ROC	Receiver-Operating Characteristic

SCT	Stress Cough Test

SUI	Stress Urinary Incontinence

TENS	Transcutaneous Electrical Nerve Stimulation

UAB	Underactive Bladder

UI	Urinary Incontinence

UUI	Urge Urinary Incontinence

VD	Vaginal Delivery

